# Epithelial-Myoepithelial Carcinoma of the Minor Salivary Glands: Case Series with Comprehensive Review

**DOI:** 10.3390/diagnostics11112124

**Published:** 2021-11-16

**Authors:** Kohei Okuyama, Yasuyuki Michi, Yoshihisa Kashima, Hirofumi Tomioka, Hideaki Hirai, Misaki Yokokawa, Yuko Yamagata, Takeshi Kuroshima, Yuriko Sato, Maiko Tsuchiya, Kou Kayamori, Tohru Ikeda, Hiroyuki Harada

**Affiliations:** 1 Department of Oral and Maxillofacial Surgery, Graduate School of Medical and Dental Sciences, Tokyo Medical and Dental University, 1-5-45, Yushima, Bunkyo-ku, Tokyo 113-8510, Japan; y-mic.mfs@tmd.ac.jp (Y.M.); kashima.osur@tmd.ac.jp (Y.K.); tomy.osur@tmd.ac.jp (H.T.); hirai.osur@tmd.ac.jp (H.H.); yokoosur@tmd.ac.jp (M.Y.); yamagata.osur@tmd.ac.jp (Y.Y.); kuroosur@tmd.ac.jp (T.K.); yurikos0803@gmail.com (Y.S.); hirosur@tmd.ac.jp (H.H.); 2 Department of Oral Pathology, Graduate School of Medical and Dental Sciences, Tokyo Medical and Dental University, 1-5-45, Yushima, Bunkyo-ku, Tokyo 113-8510, Japan; tsuchiya.maiko.yx@teikyo-u.ac.jp (M.T.); kayamori.mpa@tmd.ac.jp (K.K.); tohrupth.mpa@tmd.ac.jp (T.I.); 3 Department of Pathology, Teikyo University School of Medicine, Tokyo 173-8605, Japan

**Keywords:** epithelial-myoepithelial carcinoma, minor salivary gland, low-grade, immunohistochemistry, Ki-67, pleomorphic adenoma

## Abstract

Epithelial-myoepithelial carcinoma (EMC) is a rare salivary gland tumor that is histologically characterized by biphasic tubular structures composed of inner ductal and outer clear myoepithelial cells, which is especially uncommon in the minor salivary glands (MSG). Because of its histologic variety, complexity, and heterogeneity, it is sometimes challenging to make the accurate diagnosis. Here, we report a literature review of EMC of the MSGs with our experience of two cases. Incisional biopsy was suggestive of pleomorphic adenoma in Case 1 and pleomorphic adenoma or a low-grade salivary gland carcinoma in Case 2. Both cases were performed intraoral tumor resection, and they have good postoperative courses and are alive with no evidence of local recurrence or metastasis at 31 and 16 months, respectively. Considering that the anatomy, structure, and size of salivary glands are quite different from MSGs, it might be difficult to predict EMCs of the MSG similarly to EMCs of the major salivary glands. This comprehensive review also reports the features of EMC of the MSG cases and the trends of diagnosis and discusses treatment strategy.

## 1. Introduction

Epithelial-myoepithelial carcinoma (EMC) is a rare malignant salivary gland tumor that accounts for <1% of all salivary gland epithelial neoplasms and approximately 2% of malignant salivary gland neoplasms [1,2]. The mean age of patients with EMC at diagnosis is 60 years, and it shows a slight female predilection [3]. Most EMCs develop in the parotid gland, while some develop in the submandibular gland [1,4]. In general, EMC is indicated by the recurrence rate of 30% to 50%, lymph node metastasis rate of 15% to 20%, and 5- and 10-year survival rates of 80% to 94% and 72% to 90%, respectively [1,4,5,6]. However, these were evident in most of the major salivary gland cases. The minor salivary glands (MSG) cases are an uncommon anatomic site for the origin of EMCs. Since the primary site is very small, the bearing duration in EMC of the MSGs can inevitably be prolonged and there is difficulty in dealing with small tissue and diagnosis via multiple immunohistochemistry (IHC) staining. 

Histopathologically, it is characterized by a biphasic glandular arrangement of inner eosinophilic ductal epithelial cells and outer clear myoepithelial cells [1,2]. However, several histologic variants of EMCs, such as sebaceous [5,7], oncocytic/apocrine [8,9], and double-clear [5,10], have been described. In addition, some cases experienced high-grade malignancy, which is often associated with a poorer prognosis [1,2,5,11,12,13,14,15,16,17]. Furthermore, other benign or malignant neoplasms can exhibit clear myoepithelial features, which render the differential diagnosis of EMCs further complicated [18].

Due to its rare occurrence, the clinicopathological features and optimal treatment strategies of EMCs of the MSGs have not been fully described, and the relevant literature mostly comprises case reports. Herein, we report two cases of EMC originating from the MSG and review the relevant literature and assess our cases.

## 2. Case Presentation

### 2.1. Case 1

**Figure 1:**
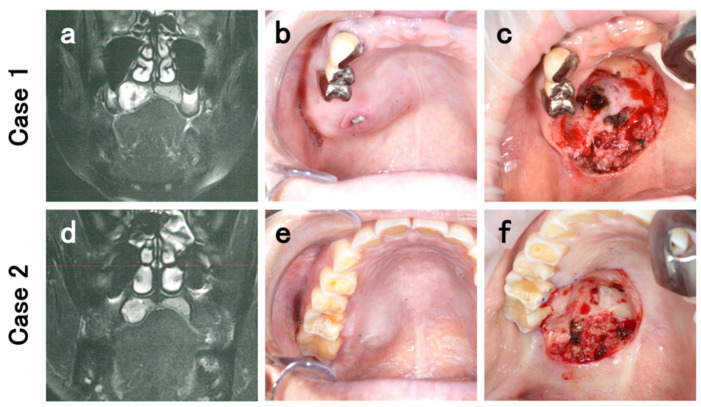
(**a**) On T2-weighted magnetic resonance imaging (MRI) shows an internal non-uniformly enhanced tumor mass, which led to pressure absorption of the palatal bone but invasion of the sinus and nasal cavity was not evident. (**b**) Intraoral view before the resection revealed a hard, elastic mass in the right side of the hard palate, sized 2.5 × 2 cm. Biopsy site was residual as an ulcerous region. (**c**) Intraoral view after the resection. Greater palatine artery and nerve were ligatured and cut. The tumor was resected including the surrounding gingiva and the periosteal, and a layer of the palatal bone was shaved off. (**d**) Similar to Case 1, T2-weighted MRI showed an internal non-uniformly enhanced tumor mass, which led to pressure absorption of the palatal bone but invasion of the sinus and nasal cavity and significant cervical adenopathy were not evident. (**e**) First intraoral examination revealed a hard, elastic mass in the right side of the hard palate, sized 1.8 × 1.6 cm and without ulceration. (**f**) Intraoral view after the resection. Greater palatine artery and nerve were ligatured and cut. The tumor was resected including the surrounding gingiva and the periosteal, and a layer of the palatal bone was shaved off.

A 75-year-old woman was referred to our department with a complaint of an abnormal swelling on the hard palate for a few months. The patient did not have any history of specific underlying systemic disease or trauma. Intraoral examination revealed a hard, elastic mass in the right side of the hard palate, sized 2.5 × 2 cm and without ulceration. Histopathological analysis of the sample obtained from incisional biopsy was suggestive of pleomorphic adenoma (PA) of the hard palate. The patient refused excision; therefore, regular follow-up visits for monitoring the tumor were performed. After a follow-up period of about 14 months, rapid tumor growth was observed. Computed tomography (CT) and magnetic resonance imaging (MRI) revealed an internal non-uniformly enhanced tumor mass, which led to pressure absorption of the palatal bone, but invasion of the sinus and nasal cavity was not evident (Figure 1a). Although the imaging examinations could not indicate significant findings of malignancy, they guided a presumptive diagnosis of malignancy, based on the intratumoral heterogeneity. Significant cervical adenopathy was not evident. At 1 year and 3 months after the first examination, tumor resection with adequate surgical margins through an intraoral approach was finally performed under general anesthesia. Intraoperatively, the tumor was resected, including the surrounding gingiva and the periosteal, and a layer of the palatal bone was shaved off. The greater palatine artery and nerve were ligated and cut (Figure 1b,c). 

### 2.2. Case 2

A 44-year-old healthy woman presented at our department with a complaint of a swelling in the hard palate. It had been followed up by her regular dentist for 2 months, but the swelling had not improved. Our intraoral examination revealed a hard, elastic mass in the right side of the hard palate, sized 1.8 × 1.6 cm and without ulceration. Histopathological analysis of the incisional biopsy sample was suggestive of PA or a low-grade salivary gland carcinoma of the hard palate. CT and MRI revealed an internal non-uniformly enhanced tumor mass, which led to pressure absorption of the palatal bone but invasion of the sinus and nasal cavity and significant cervical adenopathy were not evident (Figure 1d). Thereafter, the treatment was restricted because of the COVID-19 infection pandemic in Japan. There was no evidence of rapid tumor growth or metastasis. After four months from the first examination, since the treatment restrictions derived from the pandemic were released, intraoral tumor resection was performed under general anesthesia. Similar to Case 1, the tumor was peeled off including the periosteal and a layer of the palatal bone was shaved off. Greater palatine artery and nerve were ligatured and cut (Figure 1e,f).

Both patients have good clinical courses on surgical sites and are alive with no evidence of local recurrence or cervical lymph node / distant metastasis at 31 and 16 months after the surgery, respectively.

### 2.3. Microscopical Findings

**Figure 2:**
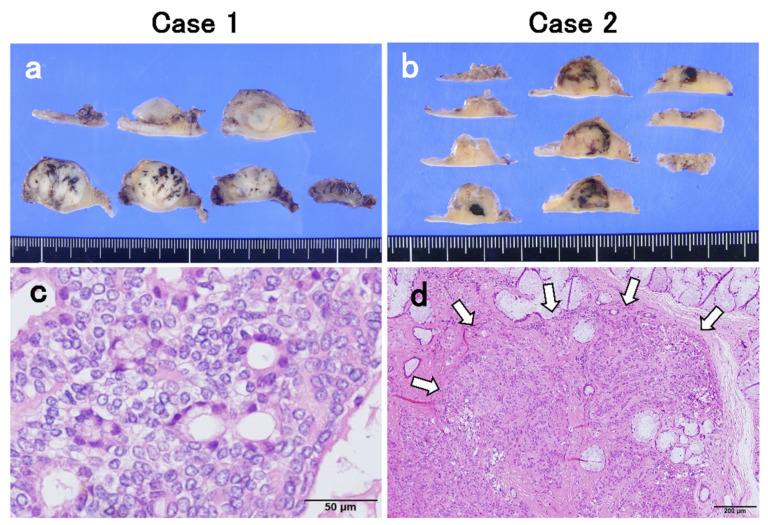
(**a**,**b**) Photographs of the specimen. Macrographically, the cut surface of the resected tumors revealed a spherical tumor, measuring 19.5 and 12.5 mm in maximum diameter, respectively. Apparent invasion findings to surrounding tissue were not detected at macro-level. Tumors included some blacky speckled structure. (**c**,**d**) Hematoxylin and eosin staining (HE) showing a component with dense growth of acidophilic tumor cells and a component with cord-like or network-like growth of epithelioid-like neoplastic myoepithelial cells, and a biphasic glandular structure consisting of glandular cavities with acidophilic vesicles and neoplastic myoepithelial cells with clear vesicles outside the cavities. Bar, 50 um. (**d**) In Case 2, although fission images are moderate, the apparent invasion to the preexisting minor salivary glands can be identified (arrows). HE staining. Bar, 200 um.

**Figure 3:**
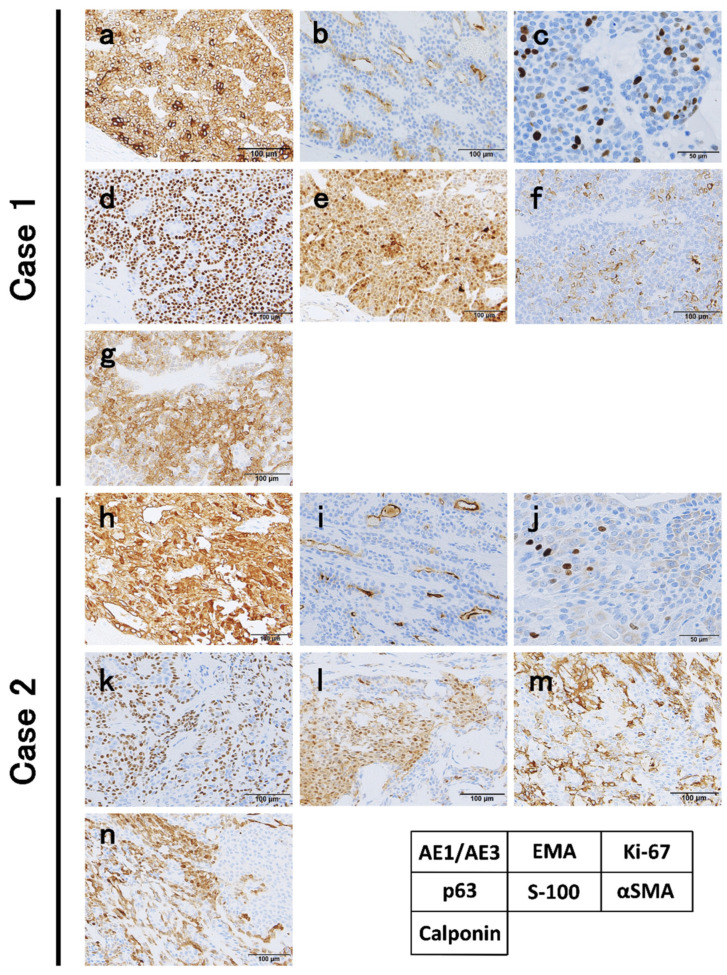
Immunohistochemical pattern of the present cases with epithelial-myoepithelial carcinoma. (**a**,**h**) The tumor was diffusely positive for AE1/AE3. (**b**,**i**) Glandular duct-forming cells were positive for epithelial membrane antigen (EMA). (**c**) Ki-67 showed active fission images and a high Ki-67 labeling index (10.6%). Neoplastic myoepithelial cells were positive for (**d**,**k**) p63, (**e**,**l**) S-100, (**f**,**m**) α-smooth muscle actin (α-SMA), and (**g**,**n**) Calponin. In case 2, (**m**) Tumor nests were diffusely positive for S-100 protein. (**j**) Ki-67 labeling index was low (3.8%).

At the macro-level, the cut surface of the resected tumor revealed a spherical tumor, measuring 19.5 and 12.5 mm in maximum diameter, respectively. Apparent invasion findings were not detected at macro-level. Tumors included some blacky speckled structures (Figure 2a,b). In our cases, a biphasic glandular structure consisting of glandular cavities with acidophilic vesicles and neoplastic myoepithelial cells with clear vesicles outside the cavities was observed under high magnification (Figure 2c,d). IHC showed that the tumor was diffusely positive for AE1/AE3; neoplastic myoepithelial cells were positive for p63, S100, calponin, and α-smooth muscle actin (α-SMA), and glandular duct-forming cells were positive for epithelial membrane antigen (EMA) (Figure 3). In Case 1, the tumor was diagnosed as EMC due to the active fission images and the high score of Ki-67 labeling index (10.6%) (Figure 3c). In Case 2, although Ki-67 labeling index score was 3.8% and fission images were not frequent (Figure 3j), as invasion into the existing MSGs was evident (Figure 2d), the tumor was diagnosed as EMC. Both cases were negative at the surgical margin.

## 3. Materials and Methods

### Review of Literature

We searched the PubMed database for English literature pertaining to EMCs of the MSGs using the keywords “epithelial-myoepithelial carcinoma” and “minor salivary gland” (www.ncbi.nlm.nih.gov/pubmed, accessed on 13 December 2020). Literature published between January 2000 and December 2020, with well-written description of disease condition, treatment, clinical course, histopathological analysis and findings including IHCs, and prognosis were considered eligible for inclusion. EMC cases in the floor of mouth were excluded because it was not clear if the primary site was the MSGs or the sublingual gland.

## 4. Results

We identified 38 cases in 27 articles about EMCs in the MSGs. Of the 38 cases, 18 cases satisfied the above-mentioned inclusion eligibility. The details are summarized in Table 1 [3,19,20,21,22,23,24,25,26,27,28,29]. The cohort included 10 women and 8 men with a median age of 64 (range: 29–83) years. The distribution of the primary subsite was as follows: the hard or soft palate in 12 patients, buccal mucosa in 4, nasal cavity in 2, oropharynx and subglottis in 1 patient, respectively. Regarding the diagnosis, while only 3 cases (3/18 cases, 16.7%) were diagnosed as EMC by incisional biopsy, other cases were pathologically diagnosed by the whole tumor examination. The primary antibodies used to diagnose EMC were almost similar: cytokeratin (CK), EMA, S100 protein, p63, calponin, CD10, CD117, vimentin, SMA, glial fibrillary acid protein, and Ki-67, and appropriate positive and negative controls were employed. The Ki-67 labeling index was calculated in 10 cases, and the median index value was 17.65% (range: 3.5–40) (Table 2). The tumor size ranged from 1 × 1 cm to 3.7 × 2.5 cm. The disease duration, from self-noticing the lesion for the first time to the visit to the hospital about the disease, ranged from 1 month to 96 months. The median postoperative follow-up period was 18.0 ± 21.2 months (range: 6–84). Adjuvant radiotherapy was performed in 5 cases (27.8%) with a positive surgical margin in 4 cases and a narrow margin in 1 case. One patient died due to local failure 48 months after tumor resection and postoperative radiotherapy.

## 5. Discussion

Some reports have highlighted the aggressive nature of EMCs. However, the prognosis indicated a low-grade malignancy of the tumor. Seethala et al. reported that 61 patients with EMC had a local recurrence rate of 36.3%, a distant metastasis rate of 5.2%, but a high five year survival rate of 93.5% [5]. Moreover, distant metastases to the lung, kidney, or brain were found in 8–l0% of the cases [30]. In the present review, local recurrence rate, distant metastasis rate, and survival rate of EMC of the MSGs were 11.8% (2/17 cases), 0% (0/17 cases), and 94.4% (17/18 cases), respectively. The cases of EMC of the MSGs had better prognosis compared with the report by Seethala et al., which included 61 EMC cases of both major salivary glands and MSGs. Vazquez et al. reported that lesions of less than 4 cm were indicative of a significantly poor prognosis [6]. However, the abovementioned reports included EMCs in the major salivary glands in a significant proportion, demonstrating a low credibility for direct application to the MSG cases. Moreover, since the anatomy, structure, and the size of major salivary glands are quite different from MSGs, the size of the tumor also cannot be directly considered as a factor to decide treatment for the MSG cases. In the present review, in fact, the tumor size of MSG cases ranged from 1 × 1 to 3.7 × 2.5 cm: their sizes were all under 4 cm. With regard to the tumor size, even though the oral cavity can be examined directly, the disease duration had a large width (range: 1–96 months), meaning that MSG disease cannot always be found earlier or more easily than disease in the major salivary glands. 

Regarding the treatment, in fact, the role of radiotherapy in EMCs of the MSGs is not discussed adequately. Adjuvant radiotherapy is recommended in major salivary gland tumors where the primary tumor is >4 cm in size or where the surgical margins are positive [31,32,33]. However, Vazquez et al. reported that the 10 year survival rate of patients treated only by surgery was 93.2% and that of those treated by surgery and adjuvant radiotherapy was 87.6% (*p* = 0.4832) [6]. The data from the present review could not validate radiotherapy (n = 5, Table 1). The role of adjuvant chemotherapy in EMC is also not well documented. According to Cerda et al., chemotherapy should be considered when irradiation is delivered in doses of 65 Gy or more in the patients with locoregional high-risk salivary gland tumors (close or positive margins) [34]. Overall, the present review could not establish consensus for the optimal treatment strategy of EMCs of the MSGs owing to the limited number of MSG cases other than surgery. The authors consider that the extent of tumor resection and the determination of safety resection margin during the initial operation are key factors to reduce tumor recurrence. Preoperative images must clarify whether the maxillary bone is invaded.

Regarding the diagnosis, the fine needle aspiration (FNA) is often used to diagnose salivary gland tumors. FNA specimens are excellent sources for molecular diagnostics because tumor cells are directly smeared without the use of DNA-damaging fixatives [35]. Several types of salivary gland tumors are characterized by pathognomonic chromosomal rearrangements, including MYB proto-oncogene (*MYB*) rearrangements in adenoid cystic carcinoma (ACC) and PA gene 1 (*PLAG1*) rearrangements in PA. Fluorescence in situ hybridization (FISH) has also been shown to be useful in identifying these rearrangements in FNA (e.g., mastermind-like transcriptional coactivator 2 [*MAML2*] in mucoepidermoid carcinoma and ETS variant 6 [*ETV6*] in secretory carcinoma) [36]. However, up to 50% of cases of ACC and PA have been shown to have intact *MYB* and *PLAG1* genes, respectively, making the specificity of gene rearrangement very high whereas the sensitivity is only modest [36,37]. In the 18 cases of our literature review, FNA and gene mutation evaluation were not performed. The authors speculate that this is because it is easy to access tumors located in the MSG compared with the parotid gland or other major salivary glands. In addition, evaluating the gene rearrangements can assist diagnosis using incisional and/or excisional biopsy specimens, but diagnosis using only FNA cannot obtain pathologically determinant information. On the other hand, the diagnostic strategy using MSG tumor specimens has basically remained unchanged: combined routine HE with multi-IHC staining (Table 2). Our cases were also stained with almost the same as other reports in this review: pan-CK, EMA, p63, S-100, α-SMA, calponin, and calculation of the Ki-67 labeling index. Seethala et al. reported that the specificity and sensitivity of p63 were excellent for labeling the myoepithelial epithelium, and the positive rate was up to 100% [5]. Some myoepithelial markers, such as vimentin and calponin, have been proved to be of good sensitivity and specificity for salivary tumor myoepithelial cells, facilitating confirmation of diagnosis and reflecting the prognosis of patients [38,39]. However, these accepted diagnostic methods do pose difficulties in the differential diagnosis of EMCs. Upon microscopic examination, PA is composed of epithelial and myoepithelial cells within variable stroma that may comprise of myxoid, fibrous, chondroid, mucinous, or even osseous/cartilage tissue: the biphasic pattern of epithelial and myoepithelial cells in this tumor resembles EMC. Most EMCs show a multinodular pattern with evidence of invasion and a classic arrangement of myoepithelial cells with clear cytoplasm that strikingly contrast with the inner low-cuboidal luminal cells. The biphasic structures of EMCs have an interface of a thickened, hyaline-like basement membrane, which is distinct from the chondromyxoid matrix intermingling with the outer myoepithelial cells in PAS staining [15]. Cases of an EMC arising in a PA have been described in the literature, and the diagnosis of malignancy in such cases is predicated on the presence of invasion [5,40,41]. According to Eneroth et al., the risk of malignancy in PA varies from 1.6 to 7.5% [42]. Our Case 1 might be this type. Moreover, an analysis of recurrent PAs indicated that 7.1% underwent malignant transformation and that the risk of malignancy increased with disease progression [43]. In general, carcinoma arising in a PA is difficult to diagnose because the mixed tumor component is often small and easily overlooked and the malignant component may be difficult to classify [44]. From this viewpoint, it can be deduced that the diagnostic accuracy of incisional biopsy is not high. In this review, only three cases (16.7%) could be diagnosed as EMC by incisional biopsy, indicating the limit of the microscopical diagnosis of EMC of the MSGs using a part of small mass. In contrast, El Hallani et al. indicated that 80% of EMCs arising in a PA and the genetic profile of patients with EMCs varied between the absence or presence of preexisting PA and its cytogenetic signature [40]. Moreover, Nakaguro and Urano et al. reported that the evaluation of RAS Q61R can be a useful tool to diagnose EMC, as IHC staining for RAS Q61R is highly sensitive and specific for detecting the HRAS Q61R mutation, which has been reported to be frequent in, and specific to, EMC [18,45]. 

## 6. Conclusions

Diagnosis of EMC of the MSG cases is still difficult, because the tumor is small and a mimicking tumor could be present. Additional other diagnostic methods (e.g., genomic analysis) may be helpful for an accurate presurgical diagnosis. The present review revealed no consensus on treatment strategy for EMC of the MSG other than surgical treatment and its prognosis is comparatively well. At the present, the extent of tumor resection and the determination of safety resection margin during the initial operation are key factors to control EMC of the MSG. More MSG cases should be accumulated to establish accurate treatment approach and to find the prognosticator.

## Figures and Tables

**Table 1:** Review of clinical characteristics of the cases with epithelial-myoepithelial carcinoma of the minor salivary glands.

Case No.	Author/Year	Age	Sex	Subsite	Size (mm)	cT	cN	Surgery	Surgical Margin Status	Adjuvant Treatment (Gy)	Local Recurrence	Metastasis	Prognosis	Disease Duration (Month)	f/u Duration (Month)
1	Wang, et al./2020	29	F	palate	n/a	1	0	R	n/a	-	-	-	Alive	n/a	84
2		41	F	oropharynx	n/a	2	0	R	n/a	-	-	-	Alive		48
3		52	M	palate	n/a	1	0	R	n/a	R (n/a)	-	-	Alive		24
4	Lee, et al./2020	75	F	nasal septum	37 × 25	2	0	R	n/a	-	-	-	Alive	36	18
5	Palaniappan, et al./2019	58	F	palate	15 × 15	1	0	PM	N	-	-	-	Alive	1	12
6	Tsuji, et al./2016	71	M	buccal	19 × 15	1	0	R	P	R (60)	-	-	Alive	48	48
7	Oh, et al./2016	78	F	subglottis	10 × 10	1	0	R	N	-	-	-	Alive	1	12
8	Sedassari, et al./2015	42	M	palate	n/a	4	0	R	P	-	-	-	Alive	n/a	18
9		56	F	palate	n/a	4	0	R	P	R (n/a)	+	-	Death		48
10		70	M	buccal	n/a	3	0	R	P	R (n/a)	+	-	Alive		12
11	Lima, et al./2012	61	M	buccal	20 × 20	2	0	R	N	-	n/a	n/a	Alive	96	n/a
12	Angiero, et al./2009	83	M	palate	15 × 15	1	0	R	N	-	-	-	Alive	n/a	15
13		58	F	palate	35 × 20	2	0	R	N	-	-	-	Alive	n/a	13
14		75	M	buccal	25 × 25	2	0	R	N	-	-	-	Alive	6	6
15	Teppo, et al./2008	53	M	h/palate	15 × 15	1	0	R	N	R (60)	-	-	Alive	n/a	54
16	Yamanegi, et al./2008	70	F	nasal cavity	36 × 30	2	0	R	N	-	-	-	Alive	3	12
17	Inoue, et al./2001	66	F	palate	20 × 20	2	0	PM	N	-	-	-	Alive	12	24
18	Li, et al./2000	72	F	palate	26 × 20	2	0	R	N	-	-	-	Alive	n/a	24
19	Present case 1./2021	75	F	palate	25 × 20	2	0	R	N	-	-	-	Alive	15	31
20	Present case 2./2021	44	F	palate	18 × 16	1	0	R	N	-	-	-	Alive	6	16

F, female; M, male; n/a, not available; R (Number), radiotherapy; R, resection; PM, partial mandibulectomy; N, negative; P, positive; m, month; y, year; f/u, follow-up; n/a, not available.

**Table 2:** Antibodies and results of immunohistochemical staining.

Case No.	CK (Details Unknown)	CK 5/6	CK 7	pan-CK	CK CAM5.2	EMA	CD 10/117	p63	CEA	Vimentin	S-100	α-SMA	Calponin	GFAP	PCNA	Actin	Laminin	Type IV Collagen	PAS	Ki-67 Labeling Index (%)
1							+	+			+	+	++							n/a
2							+	+			+	+	++							n/a
3							+	+			+	+	++							n/a
4			++					+			+									4
5		++						++				-	+							n/a
6			+		+	+		+	+	+	+	+								n/a
7	++							+				+								n/a
8	+		+	++				+		+		+								40
9	+		+	++				+		+		+								40
10	+		+	++				+		+		+								40
11				+				+			+									25
12		+	+	++		++	+	++		-	+	-	+	-						10
13		+	++	++		++	+	++		-	+	+	+	-						5
14		+	++	++		++	+	++		-	+	-	+	-						10
15	+				+	+				+	+	+	+			+				30
16	+			+	+	+														10
17	+									+	+	+		+						n/a
18	+									+	+			+	++		+	+	+	n/a
19				+		+		+			+	+	+							11
20				+		+		+			+	+	+							4

++, intensively positive in targeting cells; +, positive in targeting cells; -, negative in targeting cells; CK, cytokeratin; CD, cluster of differentiation; CEA, carcinoembryonic antigen; EMA, epithelial membrane antigen; SMA, alpha-smooth muscle actin; GFAP, glial fibrillary acidic protein; PCNA, proliferating cell nuclear antigen; PAS periodic acid-Schiff; n/a, not available.
